# The relationship between health-related quality of life, acceptance of illness and characteristics of pregnant women with hyperglycemia

**DOI:** 10.1186/s12955-020-01582-y

**Published:** 2020-10-02

**Authors:** Grażyna Iwanowicz-Palus, Marta Zarajczyk, Agnieszka Bień

**Affiliations:** Chair and Department of Development in Midwifery, Faculty of Health Sciences, Medical University of Lublin, 4-6 Staszica St., 20-081 Lublin, Poland

**Keywords:** Quality of life, Acceptance of illness, Woman, Pregnancy, Gestational diabetes mellitus

## Abstract

**Background:**

The study was performed to evaluate the association between socio-demographic factors on the one hand, and quality of life and illness acceptance on the other, in pregnant women with hyperglycemia.

**Methods:**

The study was performed in the years 2016–2017 in south-eastern Poland. The study included 676 women: 339 pregnant women with hyperglycemia in the case group, and 337 healthy pregnant women in the control group. The research instruments applied included the WHOQOL-BREF quality of life questionnaire, the Acceptance of Illness Scale (AIS), and a general questionnaire.

**Results:**

Factors associated with quality of life in women with hyperglycemia include: relationship status, residence, professional activity, living conditions, number of pregnancies, self-reported knowledge of diabetes treatment and lifestyle and also of the potential pregnancy complications and fetal health impact associated with the disease, as well as the type of diabetes treatment (*p* < 0.05).The mean illness acceptance score among the patients is near the lower boundary of “moderate”, 31.37 points. Factors associated with illness acceptance in women with hyperglycemia include: professional activity, living conditions, and self-reported knowledge of diabetes treatment and lifestyle and of the potential pregnancy complications and fetal health impact associated with the disease (*p* < 0.05).

**Conclusion:**

Better overall quality of life, general perceived health, and quality of life in all specific domains was found among healthy pregnant women compared to those with hyperglycemia. A higher level of illness acceptance has a positive effect on overall quality of life, general perceived health, and quality of life in all specific domains. General Quality of Life is positively correlated with reported living conditions and self-reported knowledge on glucose tolerance disorder treatment and lifestyle recommendations. AIS is positively correlated with living conditions, self-reported knowledge on glucose tolerance disorder treatment and lifestyle recommendations, and self-reported knowledge on possible pregnancy complications and infant health impact associated with glucose tolerance disorders.

## Background

Carbohydrate metabolism disorders in pregnant women can be associated with pregestational diabetes mellitus (PGDM), or can be first diagnosed during pregnancy. Hyperglycemia first diagnosed during pregnancy may be an indication of gestational diabetes mellitus (GDM) or diabetes in pregnancy (DIP). The distinction between GDM and DIP is based on the results of the 75 g oral glucose tolerance test (OGTT) performed during the pregnancy [1–3]. Globally, the rates of being overweight and obese are growing, which also entails an increasing prevalence of diabetes mellitus. Therefore, it is extremely important to offer prompt diagnostics for hyperglycemia to women who are pregnant or planning a pregnancy, as uncontrolled hyperglycemia may cause a number of complications related to conception, the course of pregnancy, and fetal development, and may affect the future life of the mother and her child [4–8].

A 2017 study of pregnant women by the International Diabetes Federation (IDF) showed that out of 21.3 million pregnancies complicated with hyperglycemia (16.2% of births worldwide), the woman had GDM in 86.4% of cases. Depending on the country, the estimated prevalence of gestational hyperglycemia ranges between 1% and 18.5%. These considerable differences are due to the various criteria used to diagnose and classify hyperglycemia during the pregnancy in the different regions [6, 9, 10].

Hyperglycemia in pregnancy is a major public health challenge, but it also imposes a number of lifestyle changes on the patient, potentially affecting her perceived quality of life (QoL) [4, 11].

In recent years, a number of studies on chronic illness focused on its impact on QoL. Increasing attention is paid not only to treatment methods, but also to the impact of illness on how the patient functions in physical, psychological, and environmental terms. Such a broad perspective of the patient and their condition offers a better understanding of issues related to care and treatment, indicating which ones are the most challenging for the patient [11–14].

Limitations imposed by the illness often force the patient to change their way of thinking and functioning on a daily basis. Adaptation to living with the illness, including the associated physical and psychological burden in one’s life, depends on the extent of illness acceptance. Therefore, evaluating illness acceptance provides insight into the way a patient has adapted to the demands of their condition, treatment, and lifestyle changes. Previous studies performed to identify the socio-demographic factors that affect patients’ illness acceptance allow for selecting groups of patients who particularly require assistance in various aspects of living with an illness [15–17].

Considering the major impact of QoL and illness acceptance on treatment success and patients’ psychological and physical health, in our study, we sought to identify the socio-demographic factors that could determine these two variables. Our findings might not only considerably impact care and treatment planning, but also help emphasize the psychological and social aspects of hyperglycemia in pregnant women.

## Methods

### Measures

The study was performed using the diagnostic survey method with questionnaires. The following instruments were used: the World Health Organization Quality of Life-Test Bref (WHOQOL-BREF), the Acceptance of Illness Scale (AIS), and a standardized interview questionnaire designed to record the pregnant women’s socio-demographic characteristics.

The World Health Organization Quality of Life-Test BREF (WHOQOL-BREF) allows for evaluating QoL both in healthy and ill individuals in clinical practice. It comprises 26 items referring to situations experienced by the patient in the past four weeks. Its first two items refer to the patient’s view of their overall QoL and overall health. The next four sections evaluate the respondents’ QoL in specific domains: physical or somatic, psychological, social relationships, and functioning in one’s environment. Responses are provided using a 5-item scale (1 to 5 points). The maximum score in each domain, after score transformation, is 20 points, and the minimum is 0. Higher scores in each domain reflect better of QoL reported by the respondent. The Cronbach’s α for the entire scale is 0.92 in healthy individuals, and 0.95 in ill individuals [18, 19]. The reliability of the Polish version of the questionnaire, measured by Cronbach’s α, is 0.90 for the entire scale, and between 0.69 and 0.81 for individual domains [20].

The Acceptance of Illness Scale (AIS) measures the level of illness acceptance in adult patients with any illness. It comprises 8 items describing the negative consequences of one’s condition, rated using a 5-point scale ranging from 1—completely agree to 5—completely disagree. The total score from all items (8–40 points) is used in the analysis. Higher scores indicate better adaptation to the limitations resulting from the illness. The Cronbach’s α for the Polish version is 0.85, indicating a reliability similar to that of the original, which has a Cronbach’s α of 0.82 [21].

### Ethics

The study was approved by the Medical University of Lublin Bioethics Committee (decision no. KE-0254/160/2016). Permission was also obtained from each health care institution where the study was performed. Respondents were informed that participation was anonymous, and freely provided their consent to participate. The study was performed in the years 2016–2017 in south-eastern Poland.

### Study population and recruitment

The study included 676 women: 339 pregnant women with hyperglycemia in the case group, and 337 healthy pregnant women in the control group. Pregnant women were included in the case group if they were willing to participate in the study, were 18 or older, were Caucasian, spoke Polish as a native language, had a singleton pregnancy, had been diagnosed with hyperglycemia at least 6 weeks before the study, and used health care in Poland throughout their pregnancy. Hyperglycemia diagnosis and classification followed the current guidelines issued by the Polish Diabetes Association:

- GDM was diagnosed when at least one of the following criteria was met in a 75 g OGTT: fasting glucose 92–125 mg/dL (5.1–6.9 mmol/L), glucose level at 60 min ≥ 180 mg/dL (10 mmol/L), or glucose level at 2 h 153–199 mg/dL (8.5–11 mmol/L);
- DIP was diagnosed when at least one of the following criteria was met: fasting glucose over 126 mg/dL (7 mmol/L), glucose level at 2 h in 75 g OGTT ≥ 200 mg/dL (11.1 mmol/L), or casual glucose level exceeding 200 mg/dL (11.1 mmol/L) with clinical hyperglycemic symptoms [2].

Diagnosis of other pregnancy complications, such as hypertension, thyroid disease, liver disease, imminent premature birth etc., which could affect the pregnant patients’ perception of their QoL and illness acceptance, was an exclusion criterion both for cases and for controls.

### Statistical analysis

Statistical analysis of the collected material was performed using IBM SPSS Statistics (v. 21) software. For qualitative variables, numbers and percentages in each category were given. Quantitative variables were described using means (M), standard deviations (SD), median (Me) and lower and upper quartile values (Q1 and Q3).

When there was a relatively large disproportion between the compared groups, and due to the variable measurement method (ordinal), non-parametric analysis methods were used. Comparisons between two groups were made using the Mann–Whitney U-test (Z), also called the Wilcoxon Mann–Whitney test. It is used to verify the hypothesis of no significant difference between the median values of the studied variable in two populations (assuming similar variable distributions). For comparing more than two groups, the Kruskal–Wallis ANOVA on ranks was used. Correlations between quantitative variables were analyzed using Pearson’s linear correlation coefficient (r). The Chi-squared test (χ^2^) was used to check for correlations between qualitative variables. It allows for assessing associations between variables measured on a nominal scale.

To determine the set of predictors for General Quality of Life and AIS, stepwise multiple regression was performed. The predictors included the following socio-demographic variables: age, relationship status, residence, education, professional activity, living conditions, number of pregnancies, self-reported knowledge on glucose tolerance disorder treatment and lifestyle, self-reported knowledge on the potential pregnancy complications and impact on the baby's health associated with glucose tolerance disorders, time of glucose metabolism disorder diagnosis and treatment.

Results were considered statistically significant at *p* < 0.05.

## Results

### Characteristics of cases and controls

Out of the 676 pregnant women recruited for the study, 339 (50.8%) women with gestational hyperglycemia were included as cases, and 337 (49.2%) women with uncomplicated pregnancy were included as controls.

In the case group, most respondents were aged 26–30 (31.9%), married (88.5%), living in a province capital (39.5%), holders of a master’s degree (43.1%), professionally active (61.1%), reporting good living conditions (53.1%), pregnant for the first time (37.8%); their self-reported knowledge on diabetes treatment and lifestyle (55.8%) and on potential pregnancy complications and impact on the baby’s health related to glucose tolerance disorders (51.6%) was good; they were diagnosed with hyperglycemia between the 24th and 28th week of the current pregnancy (53.4%); and they were treated with a diet and exercise regimen (36.9%).

In the control group, most women were aged 26–30 (35.0%), married (89.6%), living in rural areas (36.5%), holders of a master’s degree (44.2%), professionally active (56.7%), reporting good living conditions (52.8%), pregnant for the second time (37.7%); their self-reported knowledge on diabetes treatment and lifestyle (64.4%) and on potential pregnancy complications and impact on the baby’s health related to glucose tolerance disorders (66.7%) was average or poor (Table 1).

**Table 1:** Socio-demographic characteristics of the women in the study

Characteristics	Case group *N* (%)	Control group *N* (%)	Statistical analysis
	339 (50.8)	337 (49.2)	
Age			
18–25 y/o	60 (17.7)	91 (27.0)	*p* < 0.05
26–30 y/o	108 (31.9)	118 (35.0)	
31–35 y/o	99 (29.2)	89 (26.4)	
More than 36 y/o	72 (21.2)	39 (11.6)	
Relationship status			
Married	300 (88.5)	302 (89.6)	*p* = 0.732
Single	39 (11.5)	35 (10.4)	
Residence			
Urban—province capital	134 (39.5)	109 (32.3)	*p* = 0.076
Urban—other	106 (31.3)	105 (31.2)	
Rural	99 (29.2)	123 (36.5)	
Education			
Primary	32 (9.4)	35 (10.4)	*p* < 0.05
High school	88 (26.0)	110 (32.6)	
Vocational/college degree	73 (21.5)	43 (12.8)	
Master’s degree	146 (43.1)	149 (44.2)	
Professional activity			
Professionally active	207 (61.1)	191 (56.7)	*p* = 0.280
Professionally inactive	132 (38.9)	146 (43.3)	
Living conditions			
Very good	101 (29.8)	131 (38.9)	*p* < 0.05
Good	180 (53.1)	178 (52.8)	
Average	55 (16.2)	27 (8.0)	
Poor	3 (0.9)	1 (0.3)	
Number of pregnancies			
First pregnancy	128 (37.8)	117 (34.7)	*p* = 0.637
Second pregnancy	117 (34.5)	127 (37.7)	
Third or subsequent pregnancy	94 (27.7)	93 (27.6)	
Self-reported knowledge on glucose tolerance disorder treatment and lifestyle			
Very good	49 (14.5)	22 (6.5)	*p* < 0.05
Good	189 (55.8)	98 (29.1)	
Average/poor	101 (29.7)	217 (64.4)	
Self-reported knowledge on the potential pregnancy complications and impact on the baby's health associated with glucose tolerance disorders			
Very good	33 (9.7)	22 (6.5)	*p* < 0.05
Good	175 (51.6)	90 (26.8)	
Average/poor	131 (38.7)	225 (66.7)	
Time of glucose metabolism disorder diagnosis			
Before the current pregnancy	35 (10.3)	–	–
At first visit at the beginning of pregnancy (before week 12)	50 (14.8)	–	
Weeks 12–23	61 (18.0)	–	
At screening between weeks 24 and 28	181 (53.4)	–	
After week 28	12 (3.5)	–	
Treatment			
Diet + exercise	220 (64.9)	–	–
Diet + exercise + insulin	119 (35.1)	–	

### QoL in the case and control groups

Women with gestational hyperglycemia have a poorer overall QoL (*p* < 0.001) and perceived health (*p* < 0.001), as well as poorer QoL in all specific domains: physical (*p* = 0.004), psychological (*p* = 0.000), social (*p* < 0.001), and environmental (*p* < 0.001), compared to controls. The mean acceptance score among the hyperglycemic pregnant women was 31.37 (Table 2).

**Table 2:** QoL and illness acceptance of women in the study

WHOQOL-BREF domains	Case group					Control group					Statistical analysis	
	M	SD	Q1	Me	Q3	M	SD	Q1	Me	Q3	t	*p*
General quality of life	3.64	0.88	3.00	4.00	4.00	4.15	0.90	4.00	4.00	5.00	− 7.366	< 0.001
General health	3.43	0.83	3.00	4.00	4.00	3.98	0.81	4.00	4.00	4.00	− 8.679	< 0.001
Physical health	12.60	1.71	11.43	12.57	13.71	12.96	1.46	12.00	13.14	14.29	− 2.922	0.004
Psychological	14.92	2.36	13.33	15.33	16.67	15.90	1.93	14.67	16.00	17.33	− 5.923	0.000
Social relationships	15.21	2.52	13.33	16.00	16.00	16.03	2.27	14.67	16.00	17.33	− 4.479	< 0.001
Environment	14.88	2.35	13.50	15.00	16.50	15.64	2.03	14.50	15.50	17.00	− 4.479	< 0.001
AIS	31.37	6.38	27.00	32.00	37.00	–	–	–	–	–	–	–

M, mean; Me, median; SD, standard deviation

### QoL, illness acceptance, and socio-demographic factors

Result analysis demonstrated a link between the variables studied: QoL and illness acceptance, and socio-demographic factors (Table 3). Those pregnant women with hyperglycemia who were married reported better QoL in the psychological (*p* = 0.043) and environmental (*p* = 0.023) domains than unmarried respondents. The best overall QoL (*p* = 0.042), general perceived health (*p* = 0.042), and QoL in the environmental domain (*p* = 0.004) was found among respondents living in rural areas, compared to those living in urban areas. Women with a primary education were found to have a better overall QoL than those with other educational backgrounds (*p* = 0.032). However, QoL in the physical (*p* = 0.021), psychological (*p* = 0.039), and environmental (*p* < 0.001) domains was highest for women with a college/university education. Professionally active respondents reported better QoL in the environmental domain (*p* = 0.001). Those reporting very good living conditions had the highest scores for overall QoL, general health, and QoL in all specific domains (*p* < 0.05).

**Table 3:** Correlation analysis for QoL and illness acceptance, and the characteristics of the pregnant women with hyperglycemia who were studied

	WHOQOL-BREF Domains													
	General Quality of Life		General health		Physical health		Psychological		Social relationships		Environment		AIS	
	M	*p*	M	*p*	M	*p*	M	*p*	M	*p*	M	*p*	*M*	*p*
Age														
18–25 y/o	3.60	0.396	3.38	0.648	12.58	0.573	15.07	0.689	15.71	0.206	14.71	0.294	30.93	0.341
26–30 y/o	3.59		3.37		12.50		14.73		14.88		14.63		30.64	
31–35 y/o	3.77		3.49		12.55		15.09		15.33		15.23		31.94	
More than 36 y/o	3.57		3.49		12.85		14.85		15.11		14.90		32.07	
Marital status														
Married	3.67	0.154	3.46	0.178	12.65	0.179	15.03	0.043	15.27	0.148	15.00	0.023	31.51	0.288
Single	3.44		3.23		12.25		14.10		14.74		13.92		30.33	
Residence														
Urban—province capital	3.63	0.042	3.43	0.042	12.63	0.508	14.93	0.174	15.42	0.074	15.04	0.004	31.46	0.155
Urban—other	3.50		3.29		12.45		14.62		14.74		14.27		30.49	
Rural	3.81		3.59		12.73		15.24		15.41		15.31		32.20	
Education														
Primary	3.94	0.032	3.56	0.086	11.89	0.021	14.81	0.039	15.29	0.068	14.56	< 0.001	31.28	0.068
High school	3.45		3.27		12.19		14.45		14.85		13.90		30.10	
Vocational/college degree	3.59		3.37		12.45		14.76		14.81		14.62		31.01	
Master’s degree	3.71		3.53		12.86		15.32		15.60		15.66		32.34	
Professional activity														
Professionally active	3.65	0.753	3.45	0.572	12.71	0.148	15.08	0.118	15.42	0.050	15.21	0.001	32.07	0.011
Professionally inactive	3.62		3.40		12.43		14.67		14.87		14.36		30.28	
Self-reported living conditions														
Very good	3.93	< 0.001	3.68	< 0.001	12.98	0.001	16.09	< 0.001	15.96	< 0.001	16.24	< 0.001	33.57	< 0.001
Good	3.63		3.44		12.60		14.63		15.27		14.69		30.78	
Average/poor	3.16		2.98		11.96		13.80		13.68		13.10		29.37	
Number of pregnancies														
First pregnancy	3.73	0.327	3.52	0.294	12.78	0.293	15.41	0.008	15.72	0.013	15.24	0.073	32.17	0.145
Second pregnancy	3.62		3.39		12.44		14.77		14.94		14.74		30.57	
Third or subsequent pregnancy	3.55		3.36		12.55		14.45		14.84		14.55		31.29	
Self-reported knowledge on glucose tolerance disorder treatment and lifestyle														
Very good	3.83	< 0.001	3.67	< 0.001	13.51	< 0.001	16.27	< 0.001	16.70	< 0.001	16.32	< 0.001	34.65	< 0.001
Good	3.81		3.55		12.64		15.36		15.52		15.27		31.98	
Average/poor	3.21		3.08		12.07		13.43		13.88		13.43		28.64	
Self-reported knowledge on the potential pregnancy complications and impact on the baby's health associated with glucose tolerance disorders														
Very good	3.72	< 0.001	3.54	< 0.001	13.47	< 0.001	16.54	< 0.001	16.56	< 0.001	16.57	< 0.001	34.78	< 0.001
Good	3.82		3.57		12.88		15.47		15.47		15.43		32.24	
Average/poor	3.36		3.21		12.00		13.77		13.77		13.71		29.35	
Time of glucose metabolism disorder diagnosis														
Before the current pregnancy	3.37	0.111	3.09	0.002	12.38	0.001	14.13	0.018	14.82	0.904	14.21	0.479	30.43	0.463
At first visit at the beginning of pregnancy (before week 12)	3.58		3.42		13.14		14.81		15.44		14.58		31.78	
Weeks 12–23	3.80		3.79		13.22		15.72		15.19		15.30		32.36	
At screening between weeks 24 and 28	3.66		3.41		12.34		14.89		15.24		14.93		31.15	
After week 28	3.58		3.00		11.81		14.11		14.89		15.17		30.83	
Treatment														
Diet + exercise	3.71	0.049	3.46	0.369	12.64	0.562	15.12	0.032	15.25	0.671	15.03	0.121	31.59	0.397
Diet + exercise + insulin	3.51		3.38		12.53		14.55		15.13		14.61		30.97	

M Mean, *p* significance, AIS Acceptance of Illness Scale

The best QoL in the psychological (*p* = 0.008) and social (*p* = 0.013) domains was found for women going through their first pregnancy. The lowest level of self-reported knowledge on lifestyle and treatment for carbohydrate metabolism disorders during the pregnancy was correlated with the poorest QoL in all WHOQOL-BREF domains (*p* < 0.001). Those respondents who rated their knowledge on potential pregnancy complications and risks to the baby’s health associated with glucose tolerance disorders as “good” had a better overall QoL (*p* < 0.001) and better perceived health (*p* < 0.001). In turn, “very good” reported knowledge in this aspect was significantly correlated with better QoL in all WHOQOL-BREF domains compared to other respondents (*p* < 0.001). The poorest perceived health (*p* = 0.002) and physical (*p* = 0.001) and psychological QoL (*p* = 0.018) was reported by respondents diagnosed with hyperglycemia after week 28 of the pregnancy. Women treated with a diet and exercise plan had a better overall QoL (*p* = 0.049) and better psychological QoL (*p* = 0.032) than those also treated with insulin.

Our analysis demonstrated that professionally active respondents had a higher level of illness acceptance than those who did not work (*p* = 0.011). Moreover, pregnant women with hyperglycemia who reported very good living conditions had more illness acceptance (*p* < 0.001). Illness acceptance among women with hyperglycemia during the pregnancy also improved along with self-reported knowledge on lifestyle and treatment (*p* < 0.001) and on potential pregnancy complications and health impact (*p* < 0.001).

To determine the set of predictors for General Quality of Life and AIS, stepwise multiple regression was performed (Table 4). For General Quality of Life, the final model included 2 significant predictors, which accounted for 15% of variance in the dependent variable. The proposed regression model had a good fit to the data (F = 5.374; *p* < 0.001). The Durbin-Watson statistic was significant, and had a value of 1.853. For the AIS, the final model included 3 significant predictors, which accounted for 14% of variance in the dependent variable. The proposed regression model had a good fit to the data (F = 5.415; *p* < 0.001). The Durbin-Watson statistic was significant, and had a value of 1.812.

**Table 4:** Regression analysis for General Quality of Life, AIS, and selected statistically significant socio-demographic variables

Predictor	B	Beta	t	*p*
General quality of life				
Living conditions	0.282	0.222	3.745	0.000
Self-reported knowledge on glucose tolerance disorder treatment and lifestyle	0.260	0.199	2.598	0.010
F = 5.374; *p* < 0.001; D-W = 1.853; R^2^ = 0.149				
AIS				
Living conditions	1.080	0.118	1.984	0.048
Self-reported knowledge on glucose tolerance disorder treatment and lifestyle	1.429	0.151	1.971	0.049
Self-reported knowledge on the potential pregnancy complications and impact on the baby's health associated with glucose tolerance disorders	1.443	0.154	2.058	0.040
F = 5.415; *p* < 0.001; D-W = 1.812; R^2^ = 0.141				

Based on beta coefficients, positive correlations were identified between General Quality of Life and living conditions (beta = 0.222, *p* = 0.000) and self-reported knowledge on glucose tolerance disorder treatment and lifestyle (beta = 0.199, *p* = 0.010). AIS was positively correlated with living conditions (beta = 0.118, *p* = 0.048), self-reported knowledge on glucose tolerance disorder treatment and lifestyle (beta = 0.151, *p* = 0.049), and self-reported knowledge on possible pregnancy complications and infant health impact associated with glucose tolerance disorders (beta = 0.154, *p* = 0.040).

### Illness acceptance and QoL

Correlations between illness acceptance and QoL in women with hyperglycemia during pregnancy are shown in Table 5. QoL and illness acceptance were found to be significantly positively correlated in the respondents (*p* < 0.001). The correlations were rated at between 0.357 and 0.574. More illness acceptance was associated with better overall QoL, general health, and QoL in all specific WHOQOL-BREF domains.

**Table 5:** Correlations between illness acceptance and QoL in pregnant women with hyperglycemia

WHOQOL-BREF domains	AIS	
	r	*p*
General quality of life	0.381	< 0.001
General health	0.455	< 0.001
Physical health	0.390	< 0.001
Psychological	0.574	< 0.001
Social relationships	0.357	< 0.001
Environment	0.398	< 0.001

*AIS* Acceptance of Illness Scale

## Discussion

The diagnosis of hyperglycemia during pregnancy can affect many aspects of a woman’s life. The QoL of these women may be adversely affected by the resulting changes in mood and perceived health, and a partial loss of control over one’s own body [11, 14, 22].

Due to the continuously growing interest in patients’ QoL during illness and treatment, numerous studies have been published on the adverse impact of such conditions as type 1 or type 2 diabetes on QoL in adults and children [23–26]. However, research on the impact of hyperglycemia on the QoL of women during pregnancy is still lacking [11, 14, 27–29]. To the best of our knowledge, the present study is one of few addressing the association between illness acceptance and QoL in pregnant women with hyperglycemia.

Our findings demonstrate poorer overall QoL and perceived health in pregnant women with hyperglycemia compared to healthy pregnant women. This is consistent with other published reports [4, 14, 27]. A similar difference between healthy and diabetic women was reported in studies on women who were not pregnant [23]. In the literature, somewhat different reports can also be found, where general perceived health was lower in ill individuals than in healthy ones (similarly to our study), but overall QoL was rated higher by hyperglycemic respondents than by healthy controls [29].

In our study, pregnant women with hyperglycemia rated their overall QoL higher than their overall perceived health. Similar findings have been reported in other studies on the QoL of pregnant women [11] and non-pregnant diabetic women aged 45–55 [23]. GDM diagnosis also adversely affected perceived QoL in pregnant women in a tertiary health care center in India [14] and pregnant women in the US state of West Virginia [22].

The highest scores for specific QoL domains among hyperglycemic pregnant women in our study were found in the “social relationships” domain, which is consistent with other studies on the QoL of pregnant women and of patients with type 1 and 2 diabetes [11, 27].

In our study, the lowest-scored domain was physical QoL, as in a Brazilian study on pregnant women with diabetes or mild hyperglycemia [4]. Other researchers studying pregnant women with hyperglycemia reported the lowest scores for the psychological domain [11, 27]. Thus, research shows women with diabetes during or beyond pregnancy as individuals experiencing limitations in the performance of their social roles, resulting from their illness interfering with multiple aspects of functioning, both physical and psychological.

There are multiple factors associated with perceived QoL. In addition to the clinical symptoms and the treatment used, significant associations were found with such socio-demographic characteristics as residence, education, relationship status, and professional activity, among other factors [11, 14, 30].

In our study, pregnant women with hyperglycemia who were married reported better QoL in the psychological and environmental domains than unmarried respondents. One may hypothesize that this was due to a greater sense of security experienced by women in a legally sanctioned relationship. Similar conclusions were also reported by researchers from Columbia, who studied healthy pregnant women [30].

Residence was also associated with reported QoL. The best overall QoL, perceived health, and QoL in the environmental domain was reported by rural residents, and the poorest by women living in smaller towns. Conversely, in a study of patients with Parkinson’s disease, urban residents reported the best QoL [1].

Education is another factor associated with reported QoL in the study group. The highest overall QoL scores were found among women with a primary education, and the lowest among high school graduates. But QoL in the physical, psychological, and environmental domains was highest for women with a college/university education.

Similarly, in a study on women with type 2 diabetes in Iran [31], the best QoL in specific WHOQOL-BREF domains was reported by women who had completed higher education. Better QoL was also associated with better education in diabetic patients from Spain [12] and South Asia [32]. It seems that better education entails more confidence, security, and a better relationship with others.

In our study, professionally active women reported better QoL in the environmental domain. The positive correlation between professional activity and QoL was also reported in other studies on patients with type 2 diabetes [12, 31] or Parkinson’s disease [1]. Chronically ill patients, including pregnant women with hyperglycemia, who remain professionally active seem to have better access to information and medical care, as well as a greater sense of physical and psychological security than those who do not work.

Pregnant women reporting very good living conditions had the highest scores for overall QoL, general health and QoL in all specific WHOQOL-BREF domains. The positive correlation between the above variables was also confirmed by regression analysis. In a similar study on pregnant women with hyperglycemia, the same relationship was demonstrated for the physical, psychological and environmental QoL domains [11]. Better socio-economic status was also associated with better QoL assessed using the SF-36 questionnaire in women with GDM [14] and in healthy pregnant women [30]. Based on these findings, better living conditions seem to foster a sense of security and a better perception of oneself and one’s environment.

In our study, the best QoL in the psychological and social domains was found for women pregnant for the first time, while in a study from central Anatolia, Turkey, the highest scores were reported for women in the third trimester of the pregnancy [33]. Additional housework and tasks related to raising children, reducing the time that the woman has for herself, may be factors that impair the QoL of women going through a subsequent pregnancy [34].

In the present study, the poorest overall QoL, perceived health, and QoL in specific WHOQOL-BREF domains was found in women with the least self-reported knowledge on lifestyle and treatment in diabetes and on the associated potential for pregnancy complications and baby health impact. Based on beta coefficients from regression analysis, General Quality of Life was found positively associated with self-reported knowledge on gestational glucose tolerance disorder treatment and lifestyle.

In a study performed in another region of Poland, less knowledge on diabetes in women with GDM was likewise associated with poorer QoL [13]. According to the available reports, diabetes education provided to type 2 diabetes patients improved their QoL [9, 25]. However, in another study on women with hyperglycemia during pregnancy [11], a correlation with knowledge was only found for perceived general health. Pregnant women reporting a moderate level of knowledge on diabetes in pregnancy obtained the highest scores in this WHOQOL-BREF domain [11]. The cited findings suggest a positive impact of diabetes education on multiple aspects of hyperglycemic patients’ lives.

In our study, the poorest perceived health and QoL in the physical and psychological domains was reported by women diagnosed with glucose metabolism disorders after week 28 of the pregnancy, i.e. ones with the shortest duration of illness. Contrasting findings were reported in other studies, where QoL decreased as illness duration increased [31, 32].

Initiation of insulin therapy for diabetic patients is considered one of the three crises in the treatment process, alongside diabetes diagnosis itself and the diagnosis of complications [13]. Treatment type was also associated with QoL in our study. In Poland, carbohydrate metabolism disorders during pregnancy are treated with diet and insulin: oral hypoglycemic agents are not prescribed. Highest scores for overall QoL and psychological QoL were obtained by women treated with diet and exercise, and the lowest by those on insulin therapy. These findings are corroborated by most studies on the QoL of women with hyperglycemia in pregnancy [11, 13, 14, 28] and on QoL of non-pregnant diabetic patients [12, 14, 31]. Similar results were reported in studies on type 2 diabetes patients [24] and on pregnant women in Austria [35], though in these cases, poorer QoL was only found at the beginning of insulin therapy. The authors emphasize that the increase in reported QoL in the subsequent months of insulin therapy is associated with education provided to the patients, which reduces their fear of injections and concerns about the insulin administration technique. However, another study [25] reported no negative impact of insulin treatment on patients’ QoL.

The mean illness acceptance score in our group was 31.37 points, which was near the lower boundary of “moderate”. A comparison with reports by other authors demonstrates that the mean acceptance level was higher in hyperglycemic pregnant women than in diabetic patients [17, 26].

The analysis of our results demonstrated a link between professional activity and illness acceptance. Consistent findings were reported in another study on patients with type 2 diabetes, where professionally active respondents had a higher level of illness acceptance than those who did not work [15]. In our literature review, we also found a report that did not corroborate the correlation between illness acceptance and professional activity in patients with type 2 diabetes [17].

In previous research on pregnant women with hyperglycemia [11] and on diabetic patients [17], respondents who reported better living conditions had better illness acceptance scores. This is consistent with the present findings, where women living in better conditions were also more accepting of the illness-related restrictions in their lives.

Studies on type 2 diabetes patients [17, 36] reported an increase in illness acceptance following diabetes education. As in those studies, in our group, self-reported knowledge on lifestyle and treatment and on potential pregnancy complications and infant health impact were predictors of better illness acceptance among women with hyperglycemia during the pregnancy. Therefore, diabetes education may be considered as a component of psychological support that can alleviate the negative impact of the illness, thus promoting its acceptance. However, there are studies were diabetic patients receiving education on their illness demonstrated a lower level of illness acceptance than those who did not participate in such interventions [15]. This emphasizes the need to adapt the content and language used in education to the patients’ individual characteristics and their capacity for knowledge assimilation.

Both the present study and other literature reports on hyperglycemic pregnant women [11] demonstrate an association between illness acceptance and QoL. The higher the patient’s illness acceptance, the better her QoL. Likewise, in diabetic patients studied in Germany [16], a low level of illness acceptance was also associated with poorer reported QoL.

These findings demonstrate that patients capable of adapting to the difficulties associated with their illness have more positive thoughts, better interpersonal relationships, and a greater sense of physical and psychological security.

In summary, both the above literature review and our analyses of the association between socio-demographic characteristics and QoL and illness acceptance in pregnant women with hyperglycemia add to the available knowledge on the psychological outcomes of somatic health in pregnant patients with this condition.

Research on QoL helps identify threats and individualize the treatment and care process and provides indicators for planning and providing holistic care to pregnant women with hyperglycemia. A holistic approach to pregnant women’s health is especially important in cases of lifestyle disease, such as diabetes, which has certain psychosocial determinants. Care for women with gestational hyperglycemia should include efforts to understand their expectations, promote health education, and solve any problems arising in self-care and self-monitoring. The appropriate management of these aspects by the treatment team may help optimize obstetric care for women with hyperglycemia, and improve their QoL and illness acceptance level [11, 13, 14, 25].

## Strengths and limitations of this study

The strength of our study lies in the fact that it is one of the very few available studies on illness acceptance, QoL, and their determinants in pregnant women with hyperglycemia. It included a large group of women, lending credibility to the findings. Women with other pregnancy complications, which could affect their perceived QoL and illness acceptance, were excluded from the study.

We are also aware of certain limitations, which could provide some indications for future research. One limitation is the cross-sectional nature of the study, which precludes the establishment of any causal relationships between QoL and hyperglycemia in pregnant women. Future research could include the entire territory of Poland (as our study was only performed in its south-eastern part) and compare QoL and illness acceptance between women first diagnosed with hyperglycemia during pregnancy and those who had had diabetes before their pregnancy.

## Conclusions

Pregnant women with hyperglycemia have a poorer overall and perceived health compared to healthy pregnant women. Socio-demographic factors such as marital status, residence, education, professional activity, self-reported living conditions, number of pregnancies, self-reported knowledge on glucose tolerance disorder treatment and lifestyle, self-reported knowledge on the potential pregnancy complications and impact on the baby's health associated with glucose tolerance disorders, time of glucose metabolism disorder diagnosis and treatment used are factors associated with QoL among pregnant women with hyperglycemia.

Factors associated with illness acceptance in pregnant women with hyperglycemia include professional activity, self-reported living conditions, self-reported knowledge on glucose tolerance disorder treatment and lifestyle, self-reported knowledge on the potential pregnancy complications and impact on the baby's health associated with glucose tolerance disorders. More illness acceptance was associated with better overall QoL, general health, and QoL in all specific WHOQOL-BREF domains.

## Data Availability

The current study datasets and analysis sheets are available and will be provided due to reasonable request.

## Abbreviations

WHOQOL-BREF: World Health Organization Quality of Life-Test Bref
QoL: Quality of life
AIS: Acceptance of Illness Scale
PGDM: Pregestational diabetes mellitus
GDM: Gestational diabetes mellitus
DIP: Diabetes in pregnancy
OGTT: Oral glucose tolerance test
IDF: International Diabetes Federation
M: Mean
Me: Median
SD: Standard deviation
